# The Role of Sex in Malaria-COVID19 Coinfection and Some Associated Factors in Rivers State, Nigeria

**DOI:** 10.1155/2020/8829848

**Published:** 2020-12-02

**Authors:** E. O. Onosakponome, M. N. Wogu

**Affiliations:** ^1^Department of Medical Laboratory Science, PAMO University of Medical Sciences, Port Harcourt, Nigeria; ^2^Department of Animal and Environmental Biology, University of Port Harcourt, Nigeria

## Abstract

**Objectives:**

Data on the coinfection of malaria and COVID-19 is highly limited especially in Africa due to the novel nature of the pandemic COVID-19. Malaria and COVID-19 share striking similarities in their symptoms. A cross-sectional randomized study was conducted to investigate the role of sex in the coinfection of malaria and COVID-19 as well as some associated factors in Rivers State, Nigeria.

**Methods:**

Ethical approval was obtained from the Rivers State Health and Ethics Committee before the commencement of this study, and the study was conducted at the COVID-19 Treatment Center Medical Laboratory, Rivers State, Nigeria. Intravenous blood samples from three hundred randomly selected consenting study participants were examined for *Plasmodium* species using Giemsa microscopy, while pretested questionnaires were used to obtain data on sex, risk factors, and symptoms. All data generated were analyzed statistically using the Chi-square test with a *P* < 0.05 value considered significant.

**Results:**

All study participants had *Plasmodium* species (100% prevalence) with varying parasite loads, and *P*. *falciparum* was the only species observed. Study participants (irrespective of sex) with low and high parasitaemia had the highest and least prevalence, respectively (*P* > 0.05). Male study participants experienced more symptoms than females (*P* > 0.05) except for sore throat which had an equal value among males and females. Travel history was the only risk factor that showed significant association with sex, and males had a higher value than females (*P* < 0.05).

**Conclusion:**

Malaria and COVID-19 are major public health issues in Nigeria; more researches on these diseases especially in epidemiology, pathology, diagnosis, treatment, and vaccine production are vital.

## 1. Introduction

Malaria is a major public health issue in Africa especially in the sub-Saharan region. It is the main cause of morbidities and mortalities in young children (0-5years) and pregnant women in many developing countries [1]. Malaria is mainly transmitted through the bites of infected female *Anopheles* mosquitoes. In late 2019, China experienced an outbreak of a new beta-coronavirus with over 70,000 morbidities and 1,800 mortalities within two months. [2] The International Committee on Taxonomy of Viruses (ICTV) named the virus “SARS-CoV2” and the disease “COVID-19.” [3, 4] COVID-19 is mainly transmitted to susceptible humans through infected nasal droplets which are released via coughing, talking, or sneezing. [5] There is very little data on malaria and COVID-19 co-infection globally especially in African countries which experience both diseases with significant number of cases (morbidities and mortalities) daily. Sex (biological factor) and gender (behaviour and socio-cultural factors) are vital in understanding the dynamics of these diseases. Initial data from China and other countries globally have highlighted similar numbers of confirmed COVID-19 cases between men and women [6]. The present study was conducted to evaluate the role of sex (biological factor) in the co-infection of malaria and COVID-19 as well as some associated factors in Rivers State, Nigeria.

## 2. Materials and Methods

Before the commencement of this study, ethical clearance was obtained from the Rivers State Health and Ethics Committee. Study participants were three hundred randomly selected consenting laboratory confirmed COVID-19 patients. COVID-19 laboratory testing and confirmation was conducted prior to this study at the COVID-19 testing centres in the University of Port Harcourt Teaching Hospital (UPTH) and Rivers State University Teaching Hospital (RSUTH); both centres are located in Rivers State, Nigeria. Intravenous blood samples were collected from all study participants and examined for *Plasmodium* species using Giemsa microscopy according to standard laboratory methods [7]. Data on sex (male and female), symptoms experienced (fever, difficulty in breathing, cough/sneezing, headache, loss of smell or taste, running nose, and sore throat), and risk factors (travel history, nose mask usage, regular hand washing, and maintaining social distance) were also obtained from study participants. Malaria parasite load was graded into three groups: the high parasitaemia (HP), moderate parasitaemia (MP): and low parasitaemia (LP) groups. Data obtained were statistically analyzed by Chi-square test while Cramer's association coefficient and Fisher's exact were used to determine the goodness of fit for any significant Chi-square statistics. A *P* value less than 0.05 (*P* < 0.05) was considered significant.

## 3. Results

All study participants had *Plasmodium* species (100% prevalence) and *P*. *falciparum* was the only identified species. In Table 1, study participants with low parasitaemia (LP) had the highest prevalence of 49.7% and 57.9% in males and females, respectively, while those with high parasitaemia had the least prevalence of 21.8% and 15.7% in males and females, respectively (*P* > 0.05).

In Table 2, male study participants experienced more symptoms than females except for sore throat which had an equal value for males and females; all symptoms were not significantly associated with sex (*P* > 0.05).

Males had higher compliance to nose mask usage (58.9%), regular hand washing (58.2%), and maintaining social distance (64.2%) as well as had higher recent travel history (91.2%) (Table 3).

## 4. Discussion

All study participants had *Plasmodium* species and *P*. *falciparum* was the only species observed; some studies on malaria in Nigeria identified *P*. *falciparum* as the only malaria parasite [8–10]. Malaria is endemic in Rivers State, and it is unsurprising that all study participants had *P*. *falciparum* (despite varying parasite loads) but this also confirms that COVID-19 and malaria can be misdiagnosed in malaria-endemic areas since both diseases share similar symptoms. More research is necessary to aid in adequately distinguishing COVID-19 from malaria (vice versa) especially in rural malaria-endemic areas which lack quality laboratories or testing centres and unavailable clinicians or health personnel.

Males exhibited more symptoms than females in this study. A study conducted using 168 severe COVID-19 patients in Wuhan, China, reported that men were significantly prone to hospitalization and death as well as reduced likelihood of hospital discharge compared to women during the study [11]. In another research on SARS-CoV infection using mouse models, male mice had higher virus titres with more symptoms due to higher severe pulmonary damage from monocyte-macrophage infiltration and cytokine production [12]. More studies on co-infection of malaria and COVID-19 are needed to fully understand the role of sex (biological factor) and gender (behavioural and socio-cultural factors) in relation to susceptibility, morbidities, and mortalities.

Males had higher recent travel history and were more compliant in undertaking necessary health precautions (washing hand frequently, regular wearing of nose/face masks, and maintaining adequate social or physical distance in public places). Travelling is a major factor in the epidemiology COVID-19 and malaria; travelling or migration is a significant factor in the epidemiology of diseases globally because it plays a key role in the existence, intensity, and dissemination of diseases within a population or area. Individuals (irrespective of their sex) should minimize their public movements (except when necessary), wash their hands frequently with soap and running water or use any alcohol-based hand sanitizer, properly wear nose masks or face shields especially in public or enclosed places, and always maintain a safe distance while in a public gathering with other individuals; these will reduce the spread of COVID-19. The World Health Organization has emphasized that the response to the COVID-19 pandemic must utilize and strengthen existing infrastructure for addressing malaria and other infectious diseases globally [13]; therefore, results from this study will aid in formulating public health policies which will address the impact of COVID-19 epidemic on comorbidity, access, and control of malaria in relation to sex (biological factor).

## 5. Conclusion

Malaria and COVID-19 are major public health issues in Nigeria; more researches especially in epidemiology, co-morbidity, pathology, diagnosis, treatment, and vaccine production are vital to successfully curb the scourge of these diseases.

## Figures and Tables

**Table 1:** Prevalence of malaria and COVID-19 coinfection in relation to sex.

Sex	Number examined	Number infected (%)		
		LP	MP	HP
Male	179	89 (49.7)	51 (28.5)	39 (21.8)
Female	121	70 (57.9)	32 (26.4)	19 (15.7)
Total	300	159 (53.0)	83 (27.7)	58 (19.3)

LP: low parasitaemia; MP: moderate parasitaemia; HP: high parasitaemia.

**Table 2:** Relationship between symptoms and sex among study participants.

Symptoms	Number examined	Sex (%)	
		Male	Female
Fever			
Yes	61	38 (62.3)	23 (37.7)
No	239	141 (59.0)	98 (41.0)
Difficulty in breathing			
Yes	21	15 (71.4)	6 (28.6)
No	279	164 (58.8)	115 (41.2)
Cough/sneezing			
Yes	53	32 (60.4)	21 (39.6)
No	247	147 (59.5)	100 (40.5)
Headache			
Yes	57	35 (61.4)	22 (38.6)
No	243	144 (59.3)	99 (40.7)
Loss of smell/taste			
Yes	131	73 (55.7)	58 (44.3)
No	169	106 (62.7)	63 (37.3)
Running nose			
Yes	33	22 (66.7)	11 (33.3)
No	267	157 (58.8)	110 (41.2)
Sore throat			
Yes	14	7 (50.0)	7 (50.0)
No	286	172 (60.1)	114 (39.9)

**Table 3:** Relationship between risk factors and sex among study participants.

Risk factors	Number examined	Sex (%)	
		Male	Female
Travel history			
Yes	23	21 (91.3)	2 (8.7)
No	277	158 (57.0)	119 (43.0)
Nose mask usage			
Yes	95	56 (58.9)	39 (41.1)
No	205	123 (60.0)	82 (40.0)
Hand washing			
Yes	110	64 (58.2)	46 (41.8)
No	190	115 (60.5)	75 (39.5)
Social distance			
Yes	67	43 (64.2)	24 (35.8)
No	233	136 (58.4)	97 (41.6)

## Data Availability

Data available on request (excluding names and sensitive information of study participants due to confidentiality/ethical reasons).

## References

[1] CDC. Parasites–Malaria. https://www.cdc.gov/malaria/malari_worldwide/impact.html.

[2] Shereen M. A., Khan S., Kazmi A., Bashir N., Siddique R. (2020). COVID-19 infection: origin, transmission, and characteristics of human coronaviruses. *Journal of Advanced Research*.

[3] Lai C. C., Shih T. P., Ko W. C., Tang H. J., Hsueh P. R. (2020). Severe acute respiratory syndrome coronavirus 2 (SARS-CoV-2) and coronavirus disease-2019 (COVID-19): the epidemic and the challenges. *International Journal of Antimicrobial Agents*.

[4] Cui J., Li F., Shi Z. L. (2019). Origin and evolution of pathogenic coronaviruses. *Nature Reviews Microbiology*.

[5] Africa C. D. C. (2020). Coronavirus disease 2019 (COVID-19). Latest updates on the COVID-19 crises from Africa CDC.

[6] Klein S. L., Dhakal S., Ursin R. L., Deshpande S., Sandberg K., Mauvais-Jarvis F. (2020). Biological sex impacts COVID-19 outcomes. *PLoS Pathogens*.

[7] Cheesbrough M. (2010). *District Laboratory Practice in Tropical Countries*.

[8] Wogu M. N., Nduka F. O. (2018). Evaluating malaria prevalence using clinical diagnosis compared with microscopy and rapid diagnostic Tests in a tertiary healthcare facility in Rivers State, Nigeria. *Journal of Tropical Medicine*.

[9] AE A., B T. (2015). Prevalence of malaria parasite among asymptomatic primary school children in Angiama community, Bayelsa State, Nigeria. *Tropical Medicine and Surgery*.

[10] Pondei K., Epidor L., Eno N. (2012). Prevalence of the malaria parasite in screened blood in a tertiary health centre in the malaria – endemic Niger–Delta region of Nigeria. *Global Advance Research Journal of Microbiology*.

[11] Meng Y., Wu P., Lu W. (2020). Sex-specific clinical characteristics and prognosis of coronavirus disease-19 infection in Wuhan, China: a retrospective study of 168 severe patients. *PLoS Pathogens*.

[12] Channappanavar R., Fett C., Mack M., Ten Eyck P. P., Meyerholz D. K., Perlman S. (2017). Sex-based differences in susceptibility to severe acute respiratory syndrome coronavirus infection. *Journal of Immunology*.

[13] Ajayi I. O., Ajumobi O. O., Falade C. (2020). Malaria and COVID-19: commonalities, intersections and implications for sustaining malaria control. *The Pan African Medical Journal*.

